# Gonadotropin-releasing hormone analogue and recombinant human growth hormone treatment for idiopathic central precocious puberty in girls

**DOI:** 10.3389/fendo.2022.1085385

**Published:** 2022-12-14

**Authors:** Yuzhen Shi, Ziyi Ma, Xi Yang, Yanqin Ying, Xiaoping Luo, Ling Hou

**Affiliations:** Department of Pediatrics, Tongji Hospital, Tongji Medical College, Huazhong University of Science and Technology, Wuhan, China

**Keywords:** central precocious puberty, gonadotropin-releasing hormone analogue, recombinant human growth hormone, height, growth velocity, predicted adult height

## Abstract

**Purpose:**

To investigate the effectiveness and safety of gonadotropin-releasing hormone analogue (GnRHa) in combination with recombinant human growth hormone (rhGH) in girls with central precocious puberty (CPP).

**Methods:**

Clinical data of 80 girls diagnosed with idiopathic central precocious puberty (ICPP) between January 2017 and June 2021 were retrospectively analyzed. Treatment strategy involved GnRHa alone (group A: n=34) and GnRHa+rhGH (group B: n=46). Children’s heights (Ht), weights (Wt) and sex hormone levels were measured every 3 months after treatment and bone age (BA) every six months. Heights, growth velocity (GV), predicted adult height (PAH), weights, body mass index (BMI), sex hormone levels and bone age were compared between the two groups.

**Results:**

Children in group B showed greater height gain at the 12th, 24th and 30th months after treatment (p<0.05) than those in group A, had faster growth rates in the first and second year following treatment (p<0.05) and better PAH (p<0.05). No statistical differences in weight or BMI were found between the two groups before treatment or at any time after treatment (p>0.05). Levels of LH and FSH were lower in both groups after treatment with no statistical differences between groups (p>0.05). The gap between bone age and chronological age gradually decreased in both groups and no abnormal progression of bone age or other adverse side effects occurred.

**Conclusions:**

The combination of GnRHa with rhGH produced better height gains than GnRHa alone for patients with CPP. The gonadal axis was suppressed and progression of bone age delayed with good safety and efficacy.

## Background

The global incidence of precocious puberty in children is on the rise and is noticeably higher in girls than in boys, giving a male-to-female ratio of approximately 1:10-20 (1–3). Adolescents have had more online classes and taken less outdoor activity since the outbreak of coronavirus disease 2019 and rates of childhood overweight and obesity have increased (4). Diagnoses of precocious puberty have increased quite significantly in recent years, perhaps partly influenced in part by this (5), which prompted increased scrutiny of treatment.

Central precocious puberty (CPP) is a common pediatric endocrine disorder that causes the rapid development of internal and external genital organs and presentation of secondary sexual characteristics in girls before the age of 8 years and in boys before the age of 9 years due to early activation of the hypothalamic-pituitary-gonadal axis (HPGA). Idiopathic CPP (ICPP) is the most common form in girls while boys often have underlying pathological alterations of the central nervous system (6). HPGA activation results in estrogen exposure which accelerates bone aging and causes early epiphyseal closure. The result is a shortened time window for growth and reduced adult height (7). The goal of ICPP treatment is the inhibition of sexual development, delay of skeletal maturation to increase eventual adult height and the avoidance of psychological behavioral problems. GnRHa has been the preferred CPP medication (8). However, children receiving GnRHa therapy experience growth deceleration and sometimes may not achieve their desired height (9–11) and rhGH supplementation may improve efficacy (12).

The current study aimed to evaluate the effectiveness and safety of GnRHa combined with rhGH therapy in girls with ICPP. Outcomes of children receiving GnRHa alone and in combination with rhGH therapy were compared to identify optimal treatment.

## Materials and methods

### Participants

Clinical data of 80 girls with ICPP who were admitted to the Department of Pediatrics Tongji Hospital, Tongji Medical College, Huazhong University of Science and Technology between January 2017 and June 2021 were retrospectively analyzed. ICPP diagnosis was in accordance with the guidelines for diagnosis and treatment of precocious puberty issued by the Chinese Medical Association in 2015 (13). Diagnostic criteria were as follows: (1) girls presenting with secondary sex characteristics before the age of 8 years; (2) accelerated linear growth, defined as annual growth rate higher than the mean value for age; (3) bone maturation more than 1 year above chronological age; (4) ultrasound showing enlarged ovaries and uterus and multiple ovarian follicles of diameter >4 mm; (5) HPG axis activation confirmed by peak luteinizing hormone (LH) >5 mIU/mL in response to gonadotropin-releasing hormone (GnRH) stimulation test and LH/follicle-stimulating hormone (FSH) ratio >0.6; (6) magnetic resonance imaging evaluation of the hypothalamus and pituitary gland was performed to exclude central organic pathology.

### Methods

Participants were divided into group A (n=34) who received GnRHa alone and group B (n=46) who received GnRHa+rhGH. Patients in group B had predicted adult height (PAH) below the third percentile or less than -2 SD of genetic target height. All patients received 3.75 mg GnRHa every 4 weeks and patients in group B received an additional daily dose of 0.05-0.066 mg/kg rhGH. Treatment duration was not less than 30 months. All children were followed up every 3 months after starting treatment and measurements made of height, weight and sex hormone levels. Bone age was checked every 6 months. PAH was calculated according to the method, which was established based on Bayley-Pinneau’s method and data from the national growth survey of children in nine cities of China in the year 2005, as described previously by Liang Y et al. (14). The formula used was: PAH = measured height/percentage of current bone age height to adult height. Percentage of current bone age height to adult height refers to the ratio of the 50th percentile height corresponding to current bone age/the 50th percentile height at the age of 18. This method predicted final heights of Chinese girls with CPP who were treated with GnRHa.

### Statistical analysis

SPSS 23.0 and GraphPad Prism 8.0.2 were used for statistical analysis. Differences in clinical parameters were compared by Student’s t-test. All data are expressed as mean± standard deviation and p-value < 0.05 was considered to indicate a statistically significant result.

## Results

### General data

A total of 80 girls with CPP were enrolled, including 34 in Group A and 46 in Group B. Initial ages ranged from 6 to 10 years with the group A mean being 8.02 ± 0.83 years and group B 8.73 ± 0.94 years (p<0.001). Baseline data is presented in **Table 1**.

**Table 1:** Baseline information of girls with CPP treated with GnRHa alone or in combination with rhGH.

Variable	GroupA (n=34)	GroupB (n=46)	p-value	t-value
CA (year)	8.02 ± 0.83	8.73 ± 0.94	0.0008	3.49
BA (year)	9.24 ± 1.07	10.47 ± 1.01	<0.0001	4.93
Ht (cm)	132.20 ± 7.89	134.40 ± 7.77	0.24	1.19
Wt (kg)	30.25 ± 8.34	31.84 ± 6.05	0.36	0.93
BMI (kg/m²)	17.02 ± 2.96	17.43 ± 2.05	0.50	0.68
TH (cm)	158.00 ± 3.88	158.50 ± 3.36	0.58	0.56
PAH (cm) (range)	155.60 ± 7.26 (146–167)	150.60 ± 4.92 (139–158)	<0.01	3.23
basal LH (mIU/mL)	0.57 ± 0.73	0.61 ± 0.90	0.87	0.16
basal FSH (mIU/mL)	3.26 ± 1.96	3.27 ± 1.98	0.99	0.01

Data are reported as the mean± standard deviation.

Group A: GnRHa alone group (n=34), Group B: GnRHa+rhGH group (n=46); CA,chronological age; BA, bone age; Ht, height; Wt, weight; BMI, body mass index; TH, target height; PAH, predictive adult height; LH, luteinizing hormone; FSH, follicle-stimulating hormone.

### Height, GV and PAH

**Figure 1:**
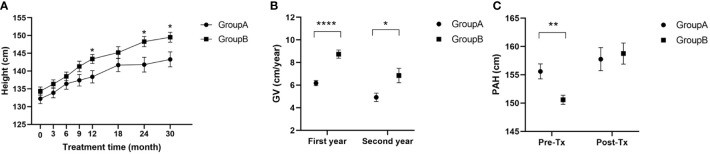
**(A)** Heights of children during treatment; **(B)** Growth velocity of children in the first and second years of treatment; **(C)** Predictive adult height of children before and after 30 months of treatment; GV, growth velocity; PAH, predictive adult height; Pre-Tx, pre-treatment; Post-Tx, post-treatment. (* p<0.05; ** p<0.01; **** p<0.0001).

No difference in mean height was present between the two groups before treatment. Heights increased for both groups after treatment and the mean was significantly greater for group B at 12, 24 and 30 months after treatment (**Figure 1A**). (A 12 months: 138.40 ± 8.63: A 24 months: 141.80 ± 7.82; A 30 months: 143.30 ± 5.16 vs. B 12 months: 143.40 ± 7.56; B 24 months: 148.30 ± 7.24; B 30 months: 149.50 ± 5.26; p<0.05). Growth rates were A: 6.18 ± 1.08 and B: 8.74 ± 2.14 (p< 0.0001, t=6.05) in the first year and A: 4.92 ± 1.41 and B: 6.85 ± 3.21 in the second year (p<0.05, t=2.59). The addition of rhGH treatment produced a remarkable advantage in growth rate (**Figure 1B**). The PAH was higher in group A (155.60 ± 7.26) than in group B (150.60 ± 4.92) before treatment (p<0.01, t=3.23). The PAH increased in both groups after treatment and there was no longer a statistically significant difference (group A: 157.80 ± 4.56 vs. group B: 158.80 ± 4.91, p>0.05, t=0.36). Thus, the addition of rhGH treatment resulted in a greater gain in PAH compared with GnRHa alone (**Figure 1C**).

**Figure 2:**
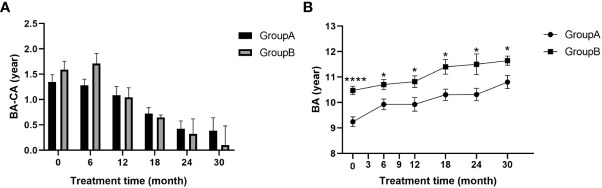
**(A)** BA-CA of children at different time points during treatment; **(B)** Bone age of children during treatment; BA, bone age; CA, chronological age. (* p<0.05; **** p<0.0001.).

### BA-CA, BA/CA and BA

There was an overall decreasing trend for the gap between bone age and chronological age (BA-CA) from the start of treatment in both groups (**Figure 2A**), indicating the effectiveness of GnRHa in slowing bone age progression, enabling the over-aged bone age to gradually converge with the child’s age. Differences between bone age and chronological age (BA-CA) (p>0.05; **Figure 2A**) and ratios of bone age to age (BA/CA) (p>0.05; **Table 2**) were not statistically different between the two groups at any time point. This suggests that addition of rhGH increased growth rate without affecting the action of GnRHa on the skeleton. There was a striking difference in bone age between the two groups before treatment (p<0.0001) and this statistically significant difference remained after 30 months of treatment (p<0.05; **Figure 2B**). Bone age increments were 1.23 ± 1.04 for group A and 0.84 ± 0.52 for group B during 30 months of treatment. Thus, addition of rhGH did not cause abnormal bone age progression.

**Table 2:** BA/CA of girls with CPP at different time points during treatment.

Treatment time(month)	0	6	12	18	24	30
BA/CA						
GroupA	1.17 ± 0.10	1.15 ± 0.07	1.12 ± 0.07	1.08 ± 0.05	1.06 ± 0.06	1.09 ± 0.11
GroupB	1.20 ± 0.12	1.19 ± 0.11	1.11 ± 0.10	1.15 ± 0.23	1.07 ± 0.10	1.04 ± 0.07
p-value	0.25	0.15	0.65	0.42	0.59	0.26
t-value	1.16	1.47	0.46	0.83	0.55	1.21

Data are reported as the mean± standard deviation.

Group A: GnRHa alone group (n=34), Group B: GnRHa+rhGH group (n=46); BA, bone age; CA, chronological age.

### Weight and BMI

**Figure 3:**
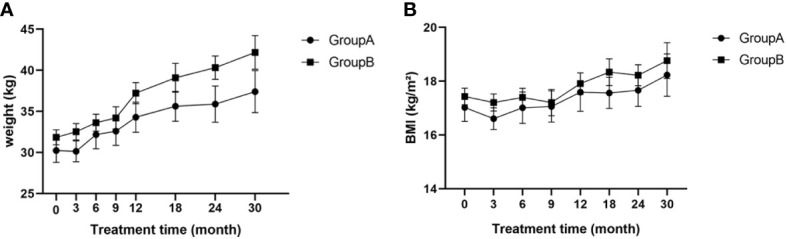
**(A)** Weight of children at different time points during treatment; **(B)** BMI of children at different time points during treatment; BMI, body mass index.

Weights of patients in both groups gradually increased throughout treatment and there were no significant differences at any time point (p>0.05; **Figure 3A**). No differences in BMI were found between the two groups at any time point and BMI remained within the normal range (p>0.05; **Figure 3B**).

### LH and FSH

**Figure 4:**
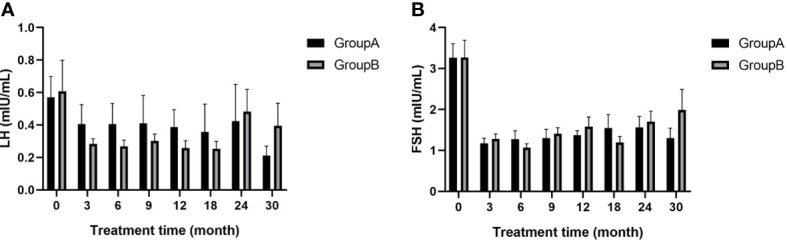
**(A)** LH of children at different time points during treatment; **(B)** FSH of children at different time points during treatment; LH, luteinizing hormone; FSH, follicle-stimulating hormone.

Basal LH and FSH levels were lower at all time points after treatment than before treatment for both groups and no significant differences were found between groups at any time point after treatment (p>0.05; **Figure 4**). This observation indicates that both treatments were effective in inhibiting gonadal axis progression.

## Discussion

Eighty patients with CPP who received GnRHa alone or GnRHa in combination with rhGH for more than 30 months were retrospectively analyzed and long-term effects evaluated. Both treatment modalities promoted height gain by suppressing the gonadal axis and slowing the progression of bone age. Furthermore, treatment with GnRHa + rhGH produced better outcomes of height, growth rate and PAH than GnRHa alone in children with CPP.

GnRHa is commonly used to treat CPP both in China and abroad. Long-acting GnRHa analogs make the gonadotrophs non-responsive since they require pulsatile stimulation by native GnRH in order to release LH and FSH. Treatment delays skeletal maturation by inhibiting sexual development to allow height catch-up and efficacy has been demonstrated in several studies (8, 15–17). However, a proportion of children with CPP did not show substantial improvement in final height or experienced growth deceleration that affected adult height outcomes after GnRHa treatment (9, 11, 18).

Exact mechanisms causing growth deceleration after GnRHa treatment are not clear but variations in estrogen levels and in the growth hormone (GH)/insulin-like growth factor 1 (IGF1) axis may be responsible. Estrogen has a dual role in bone growth, accelerating growth plate aging and promoting epiphyseal fusion while accelerating longitudinal bone growth of the growth plate and inducing pubertal growth spurts (19). Inhibition of the gonadal axis by GnRHa diminishes actions of estrogen on the growth plate which would otherwise accelerate chondrocyte proliferation. Moreover, premature exposure to estrogen in children with CPP leads to excessive senescence of the growth plate, resulting in reduced growth rate (20–23). In addition, decreased night-time GH levels and levels of biologically active IGF-I have been shown in children with CPP in response to GnRHa and the GH/IGF1 axis is central to long bone growth (24–27). Thus, the combination of GnRHa and rhGH enhances the GH-IGF-1 axis, maximizing height gain in CPP patients with severe growth deceleration or very poor PAH after GnRHa treatment.

The efficacy of GnRHa combined with rhGH in the treatment of CPP has been reported since the early 1990s (28–30). Oostdijk, W et al. (1991) found that the severely impaired growth velocity and suboptimal PAH after 3 years of treatment with GnRHa alone in CPP patients could be improved by combined growth hormone therapy (31). Pasquino AM et al. found that the gain between PAH before treatment and final height was 1.6 ± 1.2 cm in patients treated with GnRHa alone but 7.9 ± 1.1 cm for those treated with GnRHa and rhGH. Combination therapy produced a significant improvement in final adult height (12). Gains in PAH before and after treatment recorded during the current study were 8.2 ± 4.92 cm for GnRHa + rhGH and 2.2 ± 6.35 cm for GnRHa alone, showing a significant amelioration in PAH with combination therapy. Moreover, whereas PAH was significantly higher in group A than in group B before treatment, there were no differences between groups after treatment. Those with low PAH and more advanced age and BA appeared to benefit the most. The question still remains of whether rhGH was required by group B patients to achieve the final PAH or whether GnRHa was of greater benefit in more rapidly progressive CPP. A retrospective analysis of patients with idiopathic CPP showed greater height increment in the combined treatment group compared with GnRHa treatment alone. Mean height of the combination therapy group was significantly lower than that of the GnRHa alone group prior to treatment but differences were negligible after 2.1 years of treatment (32). Trends observed during the current study were consistent with those of the study by Gyon, Y. et al. stated above. No differences in height were present in the groups of the present study at the beginning of treatment and, after 1-2 years of treatment, the height in the GnRHa + rhGH group was significantly greater than that receiving GnRHa alone. The effect of combination therapy on height increase was also demonstrated in the current study. A meta-analysis of 6 randomized controlled trials and 6 case-control studies of ICPP treatment concluded that combination therapy of GnRHa and rhGH had an advantage over GnRHa alone in terms of final height but may not show a clear advantage for treatment durations of less than 1 year (33). Min Sub Kim, et al. retrospectively analyzed 166 female CPP patients treated for longer than 36 months and found that a younger age of commencement of combination therapy was associated with a higher PAH achieved (34). Therefore, an early commencement and long-term course of treatment after diagnosis are recommended with an initial treatment age of less than 10 years and duration longer than 12 months (35) to ensure maximum benefit.

Many studies have indicated advantages of combination therapy for growth velocity and PAH but the therapy has also been suggested to produce limited height improvement in children with CPP (36–38). Discrepancies could be due to several factors. There may be selection bias in the choice of study participants. For example, Jung MK et al. concluded that combination treatment did not produce a significant height improvement but mid-parental height was significantly lower for the patients treated with combination therapy than for those treated with GnRHa alone (36). Financial status may be another influential factor given the high costs associated with these treatments. In addition, nutritional status, sleep quality, exercise and other factors may also affect growth outcomes. There is a lack of data on long-term controlled clinical studies with large samples for combination therapy and a consensus has not yet been reached to recommend it as routine (39). More long-term, large-scale, prospective randomized controlled studies are required for validation.

Many reports discussing CPP combination therapy refer to only two time points at the beginning and end of treatment whereas the current study has the advantage of including multiple time points of 3, 6, 9, 12, 18, 24 and 30 months after treatment, reflecting the changes in various indicators during the dosing period more dynamically and continuously. Moreover, the focus has most frequently been on height in previous studies with fewer reports related to BMI. CPP patients treated with GnRHa had BMIs similar to those of the healthy population by late adolescence and long-term GnRHa treatment did not increase BMI (40). The current study found no statistical difference in BMI between GnRHa + rhGH and GnRHa alone at any time point (p>0.05), suggesting that combined treatment did not increase the risk of overweight or obesity.

The limitations of the present study include the following points. This was a single-center study with a relatively small sample size and, since it was retrospective in nature, patients were not randomly grouped to receive GH or not. Pre-existing baseline differences in age and bone age between the two groups were present: patients in group B were older and had more advanced BA at the start of treatment. The presence of more rapidly progressive CPP, which might mean that they responded more to the GnRHa treatment and not solely the GH, could not be excluded. Patients were not followed until the achievement of final height or growth rate <2 cm/year. However, observation of these patients will continue until they achieve their final adult height to determine the effectiveness of the combination therapy.

In conclusion, it is important to monitor the growth of patients with CPP in regular follow-up during treatment, especially when the growth rate is severely impaired and the predicted adult height is very unsatisfactory, a combination of rhGH and GnRHa therapy can be considered to gain valuable time for catching up growth.

## Data availability statement

The original contributions presented in the study are included in the article/supplementary material. Further inquiries can be directed to the corresponding authors.

## Ethics statement

The studies involving human participants were reviewed and approved by the Ethics Committee of Tongji Hospital, Tongji Medical College, Huazhong University of Science and Technology. Written informed consent to participate in this study was provided by the participants’ legal guardian/next of kin.

## Author contributions

XL and LH designed and organized the study. YS wrote the manuscript and analyzed the data. ZM and XY interpreted the data. YY collected data. LH revised the intellectual content of the manuscript. All authors contributed to the article and approved the submitted version.
